# Associations of Emotional Eating with Perceived Stress and Sleep Quality Among University Students: A Nationwide Cross-Sectional Study

**DOI:** 10.3390/nu18152434

**Published:** 2026-07-25

**Authors:** Olga Alexatou, Sousana K. Papadopoulou, Exakousti-Petroula Angelakou, Athanasios Migdanis, Maria Mentzelou, Ioannis Migdanis, Aikaterini Louka, Aspasia Serdari, Constantinos Giaginis

**Affiliations:** 1 Department of Food Science and Nutrition, University of the Aegean, 81400 Lemnos, Greece; fnsd23003@fns.aegean.gr (O.A.); xeniaggelakou@hotmail.com (E.-P.A.); maria.mentzelou@hotmail.com (M.M.); loukathy612@gmail.com (A.L.); 2 Department of Nutritional Sciences and Dietetics, International Hellenic University, 57001 Thessaloniki, Greece; souzpapa@gmail.com; 3 Nutrition and Dietetics Department, University of Thessaly, 42132 Trikala, Greece; amigdanis@gmail.com; 4 Faculty of Medicine, University of Thessaly, 41110 Larissa, Greece; imigdanis@uth.gr; 5 Department of Psychiatry, School of Medicine, Democritus University of Thrace, 68100 Alexandroupolis, Greece; aserntar@med.duth.gr

**Keywords:** emotional eating, perceived stress, sleep quality, university students, lifestyle, academic performance, anthropometric factors

## Abstract

Background/Objectives: Perceived stress and sleep quality are increasingly recognized as important determinants of emotional eating (EE), particularly among university students who are frequently exposed to academic pressures, psychosocial challenges, and lifestyle disruptions. Both stress and sleep quality are closely interconnected, with elevated stress contributing to sleep disturbances and poor sleep further exacerbating psychological distress. The present study aims to simultaneously examine the independent contributions of perceived stress and sleep quality to EE while accounting for other relevant sociodemographic, lifestyle, and anthropometric factors. Methods: This cross-sectional study was conducted among 1297 university students recruited from 10 geographical regions of Greece. Data on sociodemographic characteristics, academic profile, lifestyle behaviors, and anthropometric parameters were collected using standardized and validated assessment instruments. Perceived stress was measured using the Perceived Stress Scale (PSS), while sleep quality was evaluated with the Pittsburgh Sleep Quality Index (PSQI). Emotional eating was assessed using the Emotional Eating subscale of the Three-Factor Eating Questionnaire–Revised 18 (TFEQ-R18), and participants were subsequently classified into tertiles corresponding to low, moderate, and high levels of emotional eating. Results: Perceived stress was independently associated with EE in the multivariable analysis. Compared with students reporting low levels of stress, those experiencing high perceived stress exhibited almost threefold greater odds of being classified in higher emotional eating categories (OR = 2.86; *p* = 0.0001). Likewise, participants with moderate perceived stress were approximately twice as likely to demonstrate elevated emotional eating levels relative to their low-stress counterparts (OR = 2.01; *p* = 0.0001). Sleep quality was also independently associated with EE after adjustment for potential confounding factors. Specifically, students characterized by inadequate sleep quality had more than three times the likelihood of belonging to higher emotional eating categories compared with those reporting satisfactory sleep quality (OR = 3.08; *p* = 0.0001). The multivariable model demonstrated good fit (Likelihood Ratio Test, *p* < 0.001), and the proportional odds assumption was satisfied (*p* = 0.46). Conclusions: These findings underscore the important role of psychological distress and sleep health in EE behavior, independent of other sociodemographic, academic, lifestyle, and anthropometric factors. Given the observed associations, interventions incorporating stress-management and sleep-improvement components may represent promising approaches for addressing emotional eating among university students; however, longitudinal and intervention studies are needed to establish causality. Future longitudinal and intervention studies are needed to establish causality among perceived stress, sleep quality, and emotional eating.

## 1. Introduction

The period of transition into higher education is widely recognized as a pivotal stage of development, during which young adults encounter substantial academic responsibilities, modifications in daily routines, and numerous psychosocial challenges [1,2]. Entering university is frequently associated with increased autonomy over dietary decisions, disrupted meal schedules, and time constraints that may hinder the adoption of healthy eating practices [3]. Furthermore, adaptation to new social settings, together with financial limitations commonly experienced by students, can significantly influence food selection and eating-related behaviors [4]. Collectively, these factors may increase susceptibility to maladaptive coping responses, including problematic eating patterns. One behavior that has attracted considerable research interest is emotional eating (EE), which refers to the consumption of food in response to emotional distress rather than physiological hunger signals [5]. Individuals exhibiting EE tendencies often preferentially consume highly palatable foods rich in fat and sugar, which may offer short-term emotional comfort but are associated with unfavorable long-term health consequences [6]. Persistent reliance on food as a means of emotional regulation may eventually impair the body’s natural hunger and satiety mechanisms, thereby perpetuating unhealthy eating habits and reinforcing dysfunctional behavioral cycles [7].

University students are also considered a population at heightened risk for psychological difficulties, with studies consistently reporting elevated rates of stress, anxiety and depressive symptoms as well as poor sleep quality worldwide [8,9]. Academic workload, concerns regarding future employment opportunities, and challenges related to social integration have all been identified as important contributors to psychological distress among students [2,10]. Such emotional and psychological burdens may influence a range of health-related behaviors, with potential consequences for both physical and mental health outcomes [11]. EE has been suggested as a common coping strategy through which individuals attempt to manage adverse emotional experiences, including stress, sadness, and anxiety [5,12]. Emerging evidence indicates that this behavior may be driven, in part, by underlying neurobiological mechanisms involving reward-processing pathways and alterations in stress-responsive hormonal systems, particularly cortisol regulation [13,14]. Nevertheless, although EE may provide temporary relief from negative emotions, it may also promote unhealthy dietary patterns, increase the risk of body weight gain, and contribute to further psychological distress [14,15]. Consequently, EE appears to participate in a reciprocal relationship with mental health, functioning both as a consequence of psychological difficulties and as a factor that may exacerbate them over time [15,16].

A growing body of evidence has examined the relationship between EE, perceived stress, and sleep quality, including university students and other young adult populations [16,17,18]. Perceived stress has consistently emerged as one of the strongest psychological correlates of EE, with higher stress levels associated with an increased tendency to consume food as a coping mechanism for negative emotional states [17,19,20]. Chronic stress may promote EE through activation of the hypothalamic–pituitary–adrenal (HPA) axis and subsequent elevations in cortisol, which can increase appetite and preference for highly palatable, energy-dense foods [21,22]. Similarly, poor sleep quality has been increasingly recognized as an important factor associated with EE behaviors [18,23]. Sleep disturbances may alter appetite-regulating hormones, impair emotional regulation, and enhance reward-driven eating, thereby increasing susceptibility to emotionally triggered food consumption [24]. Several cross-sectional studies have reported that individuals experiencing inadequate sleep duration or poor sleep quality exhibit higher levels of EE compared with those reporting healthy sleep patterns [16,25]. Furthermore, emerging evidence suggests that perceived stress and sleep disturbances may interact synergistically, creating a cycle of psychological distress, impaired sleep, and maladaptive eating behaviors [26,27]. Despite these findings, relatively few studies have simultaneously investigated EE in relation to perceived stress, sleep quality, and other sociodemographic and lifestyle factors within comprehensive multivariable analytical models, particularly among university student populations in Greece.

Beyond psychological influences, EE has been linked to various sociodemographic characteristics, lifestyle habits such as sleep quality, and anthropometric parameters, including sex, levels of physical activity, smoking status, and measures of adiposity [16,18,28]. Evidence from previous research indicates that female students tend to report greater engagement in EE behaviors than their male counterparts, a finding that may be attributable to differences in emotional regulation processes and coping mechanisms between genders [29]. Moreover, unhealthy lifestyle practices, particularly low physical activity participation and tobacco use, have been associated with an increased likelihood of EE, highlighting the interconnected nature of health-related behaviors [18,19,27,28]. Anthropometric measures such as body mass index (BMI), waist circumference, and other indicators of central obesity have likewise demonstrated positive associations with EE, suggesting that this behavior may contribute to excess body weight gain and obesity risk [21,23,27,28]. Clarifying the relationships between these factors is important for the identification of vulnerable populations and the design of effective prevention and intervention strategies. Nevertheless, relatively few investigations have evaluated the independent effects of sociodemographic, lifestyle, and anthropometric characteristics concurrently with mental health factors within a single comprehensive analytical model.

Alarmingly enough, EE is increasingly recognized as a behavior of considerable public health importance because of its association with multiple adverse physical and psychological health outcomes [14,15]. Individuals who engage in EE frequently consume highly palatable foods rich in sugar, fat, and energy, often in the absence of physiological hunger. Persistent reliance on food as a coping strategy may contribute to excessive energy intake, weight gain, overweight, obesity, and abdominal adiposity. Moreover, EE has been linked to poorer dietary quality, metabolic abnormalities, and an elevated risk of developing disordered eating patterns, including binge eating behaviors [21,23]. Beyond its physical health consequences, EE has also been associated with increased psychological distress, including anxiety, depressive symptoms, reduced emotional well-being, and lower quality of life [14,16]. Given these potential consequences, the identification of factors associated with emotional eating may facilitate the development of preventive strategies aimed at improving both mental and physical health among university students.

The growing burden of EE and psychological difficulties among university students underscores the importance of clarifying the factors that contribute to these interconnected challenges. Although previous research has demonstrated that EE is influenced by a variety of behavioral and psychosocial determinants, increasing attention has been directed toward the potential roles of perceived stress and sleep quality. Elevated stress levels may promote the use of food as a coping mechanism for managing negative emotions, whereas poor sleep quality may impair emotional regulation and increase susceptibility to maladaptive eating behaviors. Despite accumulating evidence linking these factors individually to EE, their combined relationship remains insufficiently explored in university populations.

In view of the above considerations, the present cross-sectional study was designed to examine the potential associations of EE with perceived stress and sleep quality among university students. In addition, the study evaluates the contribution of sociodemographic, academic, lifestyle, and anthropometric characteristics to EE behavior. A better understanding of these relationships may facilitate the identification of vulnerable student subgroups and support the development of multidimensional prevention and intervention strategies aimed at promoting both psychological well-being and healthier lifestyle behaviors within higher education settings. To the best of our knowledge, this is the first study so far evaluating the association of EE with perceived stress and sleep quality among Greek university students.

## 2. Materials and Methods

### 2.1. Study Population

A cross-sectional design was employed for the present investigation. Initially, 1612 university students aged from 18 to 24 years old were recruited from ten geographically diverse regions across Greece, representing both metropolitan and non-metropolitan areas, including Athens, Thessaloniki, Larissa, Kalamata, Kavala, Korinthos, Alexandroupolis, Patra, and the North and South Aegean islands. Participation was limited to students who were actively enrolled in Greek universities during the study period. Data collection was conducted over a prolonged timeframe extending from March 2021 through October 2024.

The study sample was not selected through a formal probability-based sampling procedure. Instead, students were recruited from universities located in ten geographically diverse regions of Greece in an effort to achieve broad geographic coverage and include participants from both metropolitan and non-metropolitan areas. Although this approach increased the heterogeneity of the study population, it does not guarantee that the sample is fully representative of the entire Greek university student population.

Participants were recruited through voluntary participation at universities located in the ten study regions. Recruitment was conducted through direct in-person contact by trained researchers and announcements disseminated through collaborating academic departments. The same recruitment procedures were applied across all participating regions to maximize methodological consistency. Participation was entirely voluntary, and no incentives were provided. Because recruitment relied on voluntary participation rather than probability-based sampling, some degree of self-selection bias cannot be excluded.

**Figure 1:**
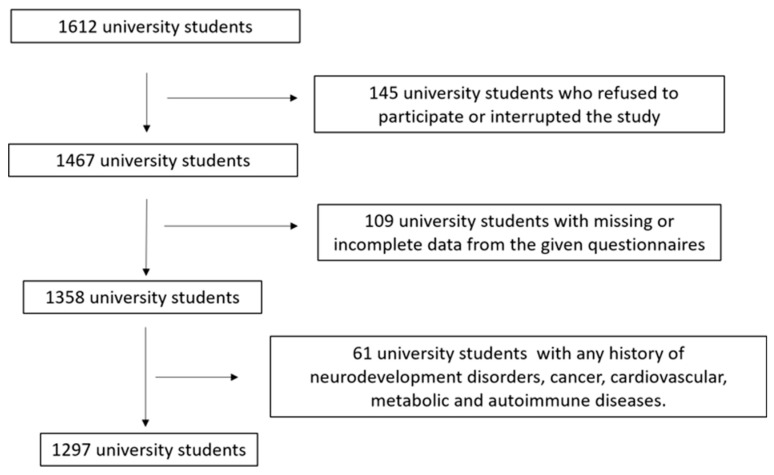
Flow chart diagram for study population enrollment.

The participant selection process is presented in Figure 1, which details the recruitment procedure and the implementation of the predetermined inclusion and exclusion criteria. Following the screening process, 1297 eligible students were included in the final analytical sample, corresponding to a response rate of 80.5%.

The required sample size was estimated prior to study initiation using the PS: Power and Sample Size Calculation software OpenEpi Version 3.01. Subsequently, a post hoc power analysis demonstrated that the final sample size achieved a statistical power of 88.1%, indicating adequate power for the primary analyses performed in the study.

The study protocol received ethical approval from the Ethics Committee of the University of the Aegean (Approval No. 21/11.10.2017; approval date: 11 October 2017) and was conducted in accordance with the ethical principles outlined in the Declaration of Helsinki as adopted by the World Medical Association (52nd WMA General Assembly, Edinburgh, Scotland, 2000). All information collected from participants was treated with strict confidentiality and used exclusively for research purposes. Prior to participation, all students were provided with detailed information regarding the objectives and procedures of the study and subsequently gave written informed consent. Eligibility criteria required participants to be free from a self-reported history of chronic medical conditions, including cardiovascular diseases, metabolic disorders, psychiatric illnesses, and malignant neoplasms, in order to minimize the potential influence of underlying health conditions on the study outcomes.

Several measures were taken to ensure that participation was completely voluntary. Before taking part in the study, students were informed that their decision to participate or not would have no effect on their grades, academic progress, relationship with faculty members, or access to university services. No financial rewards, course credits, or other incentives were offered. All questionnaires were completed anonymously, and individual responses could not be identified by instructors or university staff. Students were also informed that they could withdraw from the study at any time without providing a reason and without any consequences. These procedures were implemented to protect participants’ independence and ensure that their decision to participate was made freely.

### 2.2. Assessment of Sociodemographic, Academic, Lifestyle and Anthropometric Factors

Data regarding participants’ sociodemographic, academic, and lifestyle characteristics were collected using validated questionnaires. Information obtained included age, sex, nationality, area of residence, family income, living status, parental marital status, smoking habits, and employment status. Because all participants were enrolled in Greek universities, additional academic data were recorded, including discipline of study, duration of university attendance, and academic achievement. To ensure data quality and reduce potential recall bias, all information was gathered through structured face-to-face interviews conducted by trained nutritionists and dietitians.

Socioeconomic status was determined on the basis of annual household income and classified into three categories: low (≤10,000 €), medium (>10,000 € to ≤20,000 €), and high (>20,000 €). Academic achievement was categorized according to the official Greek university grading scale using participants’ average course grades at the time of assessment: low (5.0–6.49), moderate (6.5–8.49), and high (8.5–10.0), consistent with the standards established by the Hellenic Ministry of Education.

Physical activity was evaluated using the International Physical Activity Questionnaire (IPAQ), a widely used and validated instrument for epidemiological investigations [30]. Participants reported the frequency and duration of walking, moderate-intensity, and vigorous-intensity activities performed during a typical week. Based on established IPAQ scoring procedures, physical activity levels were classified as low, moderate, or high. Total activity was expressed as metabolic equivalent task minutes per week (MET·min·wk^−1^), calculated from all reported activities performed during the preceding seven days [30].

Anthropometric assessments were performed under standardized conditions by trained personnel. Body weight was measured to the nearest 0.1 kg using a calibrated Seca digital scale (Seca, Hanover, MD, USA), with participants wearing light clothing and no shoes. Standing height was measured without footwear using a portable stadiometer (GIMA Stadiometer 27335) and recorded to the nearest 0.1 cm [31,32]. Body mass index (BMI) was calculated as weight (kg) divided by height squared (m^2^) and categorized according to World Health Organization criteria into underweight (<18.5 kg/m^2^), normal weight (18.5–24.9 kg/m^2^), overweight (25.0–29.9 kg/m^2^), and obesity (≥30.0 kg/m^2^) [31,32]. These internationally recognized thresholds are extensively used in clinical and epidemiological research for the assessment of weight status and associated health risks.

Measures of central adiposity were also obtained, including waist circumference (WC) and waist-to-hip ratio (WHR), using standardized measurement procedures. Waist and hip circumferences were recorded to the nearest 0.1 cm. WC was categorized according to sex-specific international cut-off values: for men, normal risk (<94 cm), increased risk (94–101.9 cm), and high risk (≥102 cm); and for women, normal risk (<80 cm), increased risk (80–87.9 cm), and high risk (≥88 cm) [33]. Similarly, WHR was classified using sex-specific thresholds: for men, low risk (<0.90), moderate risk (0.90–0.99), and high risk (≥1.00); and for women, low risk (<0.80), moderate risk (0.80–0.84), and high risk (≥0.85) [33]. The application of these standardized criteria facilitates comparison across studies and enables the identification of individuals at increased cardiometabolic risk in accordance with World Health Organization recommendations [31,32].

For statistical analyses, selected continuous variables were converted into categorical variables. This approach was adopted to improve the interpretability of the findings, facilitate the application of specific analytical models, and identify clinically relevant thresholds. From both epidemiological and public health perspectives, categorization may enhance the practical utility of study results and support their translation into evidence-based recommendations and decision-making processes [34,35].

### 2.3. Assessment of Perceived Stress and Sleep Quality

Perceived stress was evaluated using the Perceived Stress Scale (PSS), a widely utilized self-report instrument designed to measure the extent to which individuals perceive situations in their lives as stressful [36]. The questionnaire consists of 10 items that assess respondents’ thoughts and feelings during the preceding month. Each item is scored on a Likert scale, with higher values reflecting greater frequency of stress-related experiences. Total scores are calculated by summing individual item responses, yielding a range from 0 to 40, with higher scores indicating greater perceived stress. Based on established cut-off values, participants were classified into three categories: low perceived stress (0–13), moderate perceived stress (14–26), and high perceived stress (27–40) [37]. Previous studies have demonstrated satisfactory reliability and validity of the PSS across diverse populations [37]. Furthermore, the Greek version of the instrument has exhibited strong psychometric performance, including high internal consistency (Cronbach’s α = 0.82) and satisfactory validity in both clinical and community samples [38]. In the present study, the PSS demonstrated good internal reliability, with a Cronbach’s alpha coefficient of 0.85.

Sleep quality was assessed using the Pittsburgh Sleep Quality Index (PSQI), a validated questionnaire consisting of 19 self-reported items that evaluate sleep characteristics over the previous month [39]. The PSQI generates seven component scores encompassing subjective sleep quality, sleep latency, sleep duration, habitual sleep efficiency, sleep disturbances, use of sleep medication, and daytime dysfunction. Each component is scored from 0 to 3, with higher scores indicating poorer sleep-related outcomes [39]. The seven component scores are summed to produce a global PSQI score ranging from 0 to 21, where increasing scores reflect progressively poorer sleep quality. According to established guidelines, a global PSQI score below 5 is considered indicative of satisfactory sleep quality, whereas higher scores suggest clinically relevant sleep impairment [39]. The Greek adaptation of the PSQI has demonstrated excellent psychometric characteristics, including very high internal consistency (Cronbach’s α = 0.985) and satisfactory validity [40]. Consistent with previous findings, the PSQI exhibited good internal reliability in the current sample, with a Cronbach’s alpha coefficient of 0.84, supporting its suitability for evaluating sleep quality among university students.

### 2.4. Emotional Eating (EE) Evaluation

Eating-related behaviors were assessed using the Three-Factor Eating Questionnaire–Revised 18 (TFEQ-R18), a validated instrument developed to evaluate key dimensions of eating behavior [41,42]. The questionnaire encompasses three distinct domains: (i) Cognitive Restraint, representing the intentional regulation and restriction of food intake for weight management purposes; (ii) Uncontrolled Eating, reflecting a tendency toward overeating accompanied by a perceived lack of control when exposed to internal sensations or external food cues; and (iii) EE, which assesses the inclination to consume food in response to negative emotional experiences [41,42]. For each dimension, higher scores correspond to a greater expression of the respective eating behavior.

The present study focused specifically on the EE domain of the TFEQ-R18. To enhance the interpretability of the findings and facilitate comparisons between groups, EE scores were categorized into tertiles representing low, moderate, and high levels of EE. The EE subscale consists of three items, producing raw scores ranging from 3 to 12. In accordance with standard scoring procedures, raw scores were converted to a standardized 0–100 scale using the following transformation formula: Score = (S − L) × 100/R, where S: individual raw subscale score, L: lowest possible raw score on that subscale, and R: possible raw score range (maximum − minimum).

The TFEQ-R18 is a widely used instrument for the assessment of eating behaviors and has exhibited robust psychometric properties in numerous population groups. Evidence from validation studies has consistently supported its underlying three-factor structure through confirmatory factor analysis, confirming the distinct dimensions of cognitive restraint, uncontrolled eating, and EE [41,42]. Furthermore, the questionnaire has demonstrated satisfactory to excellent internal reliability, with Cronbach’s alpha coefficients generally ranging between 0.70 and 0.90 for the individual subscales. Previous investigations have also reported adequate test–retest reliability, indicating that the instrument provides stable and reproducible measurements over time [41,42].

The Greek adaptation of the TFEQ-R18 has likewise undergone psychometric evaluation and has been shown to possess acceptable reliability and validity [43]. Validation studies have reported Cronbach’s alpha values exceeding 0.70 for the majority of subscales, together with satisfactory construct validity and favorable goodness-of-fit indices derived from confirmatory factor analyses [43]. These findings support the suitability of the Greek version of the TFEQ-R18 for assessing eating behavior patterns in Greek-speaking populations and justify its application in the present study.

In addition to demonstrating satisfactory internal consistency, validation studies of the Greek versions of the PSS, PSQI, and TFEQ-R18 have reported acceptable construct validity, factor structure, and convergent validity, supporting their suitability for measuring perceived stress, sleep quality, and emotional eating in Greek populations [38,40,43].

### 2.5. Statistical Analysis

Prior to statistical analyses, the distribution of continuous variables was evaluated using the Kolmogorov–Smirnov test. Variables exhibiting normality were summarized as means and standard deviations (mean ± SD) and compared between groups using Student’s t-test. For cross-tabulation analyses, categorical variables are presented as frequencies and row percentages to facilitate interpretation of the prevalence of EE categories within each subgroup, with between-group differences assessed using the chi-square test.

Scores derived from the EE subscale of the Three-Factor Eating Questionnaire–Revised 18 (TFEQ-R18) were computed according to the recommended scoring guidelines. Because the distribution of EE scores deviated from normality and to enhance the clinical and epidemiological interpretation of the findings, participants were classified into tertiles corresponding to low, moderate, and high EE levels. This categorization facilitated group comparisons and allowed the examination of potential graded associations between EE and the variables of interest.

To identify factors independently associated with EE, a multivariable ordinal logistic regression analysis was performed using EE tertiles as the dependent variable. Sociodemographic, academic, anthropometric, and lifestyle characteristics, together with perceived stress and sleep quality, were entered simultaneously into the model to account for potential confounding influences. Associations are reported as odds ratios (ORs) and corresponding 95% confidence intervals (CIs). In the multivariable ordinal logistic regression analysis, only the variables showed statistical significance in univariate analysis were included.

Prior to model development, potential multicollinearity among explanatory variables was examined using variance inflation factors (VIFs). A threshold of VIF > 5 was considered indicative of substantial collinearity; however, no evidence of problematic multicollinearity was identified. Missing observations were addressed through complete-case analysis because the proportion of missing data was minimal (<5%) and deemed to be missing at random.

The proportional odds assumption required for ordinal logistic regression was evaluated using the Test of Parallel Lines. In addition, the overall adequacy and statistical significance of the regression model were assessed through the Likelihood Ratio Test.

The final sample of 1297 participants was considered adequate for the planned multivariable ordinal logistic regression analyses. Sample size considerations were informed by the recommendations of Peduzzi et al. (1996) [44], which suggest a minimum number of observations per estimated parameter in regression models. Although this recommendation was originally developed for binary logistic regression, the large sample size available in the present study substantially exceeded these minimum requirements. Furthermore, the adequacy of the sample for ordinal regression was supported by the recommendations of Whitehead (1993) [45], which indicate that the required sample size depends on the number of outcome categories, the anticipated effect size, and the distribution of participants across categories. Given the approximately equal distribution of participants across the three emotional eating categories (*n* ≈ 432 per category), the present sample was considered sufficient to provide stable parameter estimates and adequate statistical power.

Because post hoc power calculations have been criticized as providing limited information beyond that already contained in the observed effect estimates and *p*-values [46,47], we relied primarily on the a priori sample size calculation and on methodological recommendations for ordinal regression models when evaluating sample adequacy. With this regard, the required sample size was estimated prior to study initiation using the PS: Power and Sample Size Calculation software. In addition, the adequacy of the final sample for the planned ordinal logistic regression analyses was evaluated according to methodological recommendations for ordered categorical outcomes under the proportional odds model [44]. The final analytical sample included 1297 participants distributed almost equally across the three emotional eating categories (433, 432, and 432 participants, respectively), providing a large and balanced dataset for multivariable modelling. This sample size was considered sufficient to ensure stable parameter estimates and adequate precision of the regression coefficients.

All statistical analyses were conducted using two-sided tests, and statistical significance was defined as a *p*-value below 0.05. Data management and analyses were performed using Statistica software (version 10.0; Informer Technologies Inc., Hamburg, Germany).

## 3. Results

### 3.1. Descriptive Statistics of the Study Population

The mean age of the participating students was 20.5 ± 2.5 years (age range: 18–24 years). The mean TFEQ EE subscale of the study population was 34.8 ± 21.5. The descriptive statistics of the enrolled study population are included in Table 1. It should be noted that the category “other nationality” included students originating from various countries who were enrolled in Greek universities. Unfortunately, detailed information regarding specific countries of origin was not systematically collected, preventing further stratification of this heterogeneous group.

### 3.2. Association of Emotional Eating (EE) with Sociodemographic, Academic, Lifestyle, Anthropometric, and Behavioral Factors

Cross-tabulation analyses demonstrated multiple significant relationships between EE levels and participants’ sociodemographic, academic, lifestyle, and anthropometric characteristics. Female students were more likely to report higher levels of EE than male students (Table 2, *p* < 0.0001). Similarly, participants of Greek nationality exhibited significantly greater EE scores compared with students of other nationalities (Table 2, *p* = 0.0001). A significant association was also observed with place of permanent residence, as students originating from rural areas reported higher levels of EE than their urban counterparts (Table 2, *p* < 0.0001). In addition, smoking status was significantly related to EE, with regular smokers displaying a greater prevalence of moderate-to-high EE compared with non-smokers (Table 2, *p* < 0.0001).

Several academic characteristics were also associated with EE behavior. Students enrolled in biomedical science programs demonstrated significantly higher EE levels than those studying in other academic disciplines (Table 2, *p* < 0.0001). Academic achievement was inversely related to EE, with students reporting lower academic performance exhibiting higher EE levels (Table 2, *p* < 0.0001). Moreover, participants in the more advanced years of their studies were significantly more likely to report lower EE than those in earlier stages of their academic education (Table 2, *p* < 0.0001).

Significant associations were additionally identified between EE and several lifestyle and anthropometric indicators. Students with lower physical activity levels were characterized by substantially higher EE levels than their more physically active peers (Table 2, *p* < 0.0001). Body weight status was also related to EE, with overweight and obese participants exhibiting greater EE levels compared with individuals of normal weight (Table 2, *p* < 0.0001). Likewise, indicators of abdominal adiposity, including WC and WHR, were positively associated with EE, as participants classified in higher-risk categories demonstrated significantly greater EE levels (Table 2, *p* < 0.0001 for both measures).

Behavioral characteristics emerged as important correlates of emotional eating (EE). Higher levels of perceived stress were associated with significantly greater EE levels, with students experiencing elevated stress demonstrating a higher prevalence of emotional eating compared with those reporting lower stress levels (Table 2, *p* < 0.0001). Likewise, inadequate sleep quality was strongly associated with increased EE, as participants with poor sleep quality exhibited significantly higher EE levels than their peers with adequate sleep quality (Table 2, *p* < 0.0001).

Conversely, no significant differences in EE levels were observed according to age, household income, living arrangements, parental marital status, or employment status, as all corresponding associations failed to reach statistical significance (Table 2, *p* > 0.05).

### 3.3. Multivariate Ordinal Logistic Regression Analysis for Emotional Eating (EE) Behavior of the Study Population

The multivariable ordinal logistic regression model identified several factors that were independently associated with EE levels after adjustment for potential confounders (Table 3; *p* < 0.05 for all significant associations). Female students were significantly more likely to belong to higher EE categories than male students, with an approximately 43% increase in the odds of elevated EE (OR = 1.43, 95% CI: 1.12–1.83, *p* = 0.0043). Similarly, participants of Greek nationality demonstrated higher EE levels than non-Greek students, corresponding to a 18% increase in the likelihood of belonging to a higher EE category (OR = 1.18, 95%: 1.06–1.31, *p* = 0.0022).

Place of residence also emerged as an important determinant of EE. Students originating from rural areas exhibited significantly greater odds of elevated EE compared with those residing in urban settings, with a 53% increase in the probability of belonging to higher EE categories (OR = 1.53, 95% CI: 1.19–1.97, *p* = 0.0009). Regarding academic characteristics, enrollment in biomedical science programs remained independently associated with EE (OR = 1.23, 95% CI: 1.07–1.39, *p* = 0.0046), while students in earlier stages of study were more likely to report higher EE levels than those in later academic stages (OR = 1.22, 95% CI: 1.07–1.39, *p* = 0.0032).

Among lifestyle-related factors, smoking status demonstrated a strong positive association with EE. Regular smokers exhibited a 68% greater likelihood of belonging to higher EE categories compared with non-smokers (OR = 1.68, 95% CI: 1.27–2.23, *p* = 0.0003). Moreover, students reporting low levels of physical activity had significantly higher odds of elevated EE, corresponding to a 36% increase compared with participants characterized by medium physical activity levels (OR = 1.38, 95% CI: 1.09–1.75, *p* = 0.0078). Accordingly, students reporting low physical activity levels had more than twice the odds of reporting higher EE than those reporting high physical activity levels (OR = 2.12, 95% CI: 1.40–3.21, *p* = 0.0004).

Anthropometric measures were consistently associated with EE. Students classified as obese according to BMI criteria had more than twice the odds of reporting higher EE levels relative to normal-weight participants (OR = 2.10, 95% CI: 1.45–3.05, *p* = 0.0001). Likewise, overweight individuals exhibited nearly double the likelihood of elevated EE compared with those of normal weight (OR = 1.95, 95% CI: 1.33–2.87, *p* = 0.0007).

Similar findings were observed for indicators of abdominal adiposity. Participants categorized as high risk based on WC demonstrated almost twofold greater odds of belonging to higher EE categories than those with normal WC values (OR = 1.93, 95% CI: 1.33–2.80, *p* = 0.0005). A comparable association was identified for WHR, with students classified in the high-risk category showing approximately twofold greater odds of elevated EE compared with individuals presenting normal WHR values (OR = 2.05, 95% CI: 1.44–2.93, *p* = 0.0001).

Psychological and sleep-related factors exhibited some of the strongest associations with EE. Students reporting high levels of perceived stress were nearly three times more likely to belong to higher EE categories than those experiencing low stress levels (OR = 2.86, 95% CI: 1.68–4.85, *p* = 0.0001). Similarly, moderate perceived stress was associated with approximately twofold higher odds of elevated EE compared with low perceived stress (OR = 2.01, 95% CI: 1.41–2.87, *p* = 0.0001). Sleep quality was also independently related to EE, as participants with poor sleep quality demonstrated approximately threefold greater odds of belonging to higher EE categories than those reporting adequate sleep quality (OR = 3.08, 95% CI: 1.75–5.42, *p* = 0.0001).

No significant associations were observed for the intermediate categories of central adiposity, including increased-risk WC compared with normal WC and moderate-risk WHR compared with normal WHR (*p* > 0.05). Furthermore, academic performance did not remain a significant predictor of EE after adjustment for the other variables included in the model (Table 3, *p* > 0.05).

Diagnostic testing confirmed the adequacy of the ordinal logistic regression model. The proportional odds assumption was satisfied, as indicated by the non-significant Test of Parallel Lines (*p* = 0.46), while the Likelihood Ratio Test demonstrated that the overall model provided a statistically significant fit to the data (*p* < 0.001).

### 3.4. Sensitivity Analyses Using Continuous EE, Perceived Stress, and Sleep Quality Scores

To evaluate whether the observed associations were influenced by the categorization of emotional eating into tertiles, additional sensitivity analyses were conducted using the continuous EE score derived from the TFEQ-R18 (Table S1). Since the distributions of the EE, perceived stress, and sleep quality scores deviated from normality, Spearman rank correlation analyses were performed. EE scores were positively correlated with perceived stress scores (Spearman’s ρ = 0.46, *p* < 0.0001), indicating that students reporting higher levels of perceived stress tended to exhibit greater EE tendencies. Similarly, EE scores demonstrated a significant positive correlation with PSQI scores (Spearman’s ρ = 0.41, *p* < 0.0001), suggesting that poorer sleep quality was associated with higher EE levels. In addition, perceived stress and sleep quality scores were significantly correlated with one another (Spearman’s ρ = 0.43, *p* < 0.0001), indicating that higher perceived stress was associated with poorer sleep quality. These findings were consistent with those obtained from the ordinal logistic regression model using EE tertiles. The results of these additional analyses have been included in the Supplementary Material (Table S1).

As a sensitivity analysis, a multiple linear regression model was additionally performed using the continuous TFEQ-R18 emotional eating score as the dependent variable. As independent variables PSS score and PSQI score were used, including simultaneously all sociodemographic, academic, lifestyle, and anthropometric variables (Table S2). The overall regression model was statistically significant (F = 42.6, *p* < 0.001), explaining approximately 39% of the variance in EE scores (adjusted R^2^ = 0.38). Higher perceived stress (β = 0.40, *p* < 0.001) and poorer sleep quality (β = 0.29, *p* < 0.001) remained independently associated with higher EE scores after adjustment for sociodemographic, academic, lifestyle, and anthropometric factors. Specifically, each one-point increase in the PSS score was associated with a 0.86-point increase in emotional eating score (β = 0.40, *p* < 0.001), whereas each one-point increase in the PSQI score was associated with a 1.15-point increase in EEA score (β = 0.29, *p* < 0.001). Overall, the results of the multiple linear regression analysis were consistent with those obtained from the ordinal logistic regression model, supporting the robustness of the observed associations irrespective of whether EE was analyzed as a continuous or categorical outcome variable.

## 4. Discussion

The present study showed that both elevated perceived stress and poor sleep quality were associated with higher levels of EE, independent of the covariates included in the multivariable model. Moreover, the magnitude of these associations exceeded that observed for several other established correlates of EE, highlighting the potential importance of psychological and behavioral factors in shaping eating behaviors during young adulthood.

The observed association between perceived stress and EE is consistent with recent evidence from university student populations worldwide. Several contemporary studies have identified perceived stress as one of the strongest psychological correlates of EE, with stressed individuals showing a greater tendency to consume food in response to negative emotional states rather than physiological hunger [48,49,50,51,52]. Similarly, Carpio-Arias et al. (2022) [53] and El-Zayat et al. (2025) [17] reported significant positive associations between perceived stress and EE among university students. The magnitude of the association observed in the present study is comparable to that reported in several recent investigations, although Hanbazaza et al. (2026) found even stronger associations between severe academic stress and EE after adjustment for multiple confounders [54]. Differences in stress assessment methods, population characteristics, and analytical approaches may partly explain these discrepancies. Nevertheless, the consistency of findings across diverse populations supports the hypothesis that psychological stress constitutes a major determinant of emotional eating behavior among young adults.

Furthermore, the independent association between poor sleep quality and EE observed in the present study is likewise supported by an expanding body of recent literature. Several previous studies have shown that poor sleep quality, insufficient sleep duration, and sleep disturbances are associated with greater EE, increased consumption of energy-dense foods, and impaired appetite regulation [48,55,56,57,58,59,60,61]. For example, Priede et al. (2025) [23] and Zhou et al. (2024) [59] reported that sleep quality remained a significant predictor of EE among university students, even when psychological factors were considered. Similarly, Strete et al. (2025) showed that both academic stress and sleep quality independently predicted EE among university students [27].

Compared with the above previous studies, the present analysis provides particularly robust evidence because sleep quality remained independently associated with EE after simultaneous adjustment for perceived stress, physical activity, smoking, obesity-related anthropometric measures, academic characteristics, and other potential confounders. These findings are consistent with the hypothesis that sleep disturbances may be associated with EE through alterations in appetite regulation and emotional processing. In many earlier studies, sleep quality was examined either independently or within relatively limited analytical frameworks that did not adequately account for important confounding factors, including obesity, physical activity, smoking behavior, and psychological distress [16,17,48,60,61,62]. Consequently, most of the previous studies were characterized by uncertainty regarding whether the observed association between sleep and EE reflected a direct relationship or was attributable to other coexisting behavioral and psychological factors. On the other hand, the present findings provide evidence that poor sleep quality contributes independently to EE beyond its well-established association with stress and other health-related behaviors. This observation is particularly noteworthy because relatively few studies have simultaneously examined perceived stress, sleep quality, anthropometric measures, and lifestyle behaviors within a single multivariable analytical model.

Moreover, some differences in the magnitude of associations have been reported across previous studies. Notably, Hanbazaza et al. (2026) [54] found that severe academic stress was associated with more than sixfold higher adjusted odds of severe EE, representing a substantially stronger relationship than that observed in the present study. Similarly, recent studies conducted among university students during and following the COVID-19 pandemic recorded particularly strong associations between psychological distress, perceived stress, and EE, likely reflecting the heightened emotional burden experienced during these periods [17,48,50]. Several methodological and contextual factors may explain these discrepancies. First, previous investigations frequently focused on specific stress domains, such as academic stress or pandemic-related stress, whereas the present study assessed overall perceived stress. Stressors directly related to academic performance may exert a more immediate influence on eating behavior among university students, thereby producing stronger effect estimates. Second, variations in EE assessment instruments, categorization methods, and population characteristics may contribute to differences in observed associations [47,48]. Third, the present study simultaneously adjusted for a broad range of sociodemographic, lifestyle, academic, anthropometric, and psychological variables, which may have resulted in more conservative estimates of the independent effect of stress. Importantly, despite these adjustments, perceived stress remained one of the strongest predictors of EE, reinforcing findings from recent studies that identify psychological stress as a major determinant of emotionally driven eating behavior among young adults [16,17,24,25,51,53].

Another notable finding concerns the comparative magnitude of the associations observed. High perceived stress (OR = 2.86) and poor sleep quality (OR = 3.08) demonstrated substantially stronger relationships with EE than most other variables included in the model. The magnitude of these associations exceeded those observed for female sex, smoking status, academic discipline, and year of study, and was comparable to or greater than the associations identified for obesity-related anthropometric indicators. These findings are broadly consistent with recent studies suggesting that psychological distress and sleep disturbances may represent more proximal determinants of EE than demographic or academic characteristics [50,60,62]. While previous research has frequently emphasized the contribution of obesity and lifestyle factors to EE, the present results suggest that emotional and behavioral regulation processes may occupy a more central position in the development of emotionally driven eating among university students. The comparatively large effect sizes observed for stress and sleep quality further highlight the potential importance of these factors as primary targets for preventive interventions.

The present findings extend previous literature by showing that perceived stress and sleep quality remained significantly associated with EE in multivariable analyses, suggesting that both factors may contribute to EE behavior through partially distinct pathways. Recent studies have increasingly suggested that sleep disturbances may mediate the relationship between stress and maladaptive eating behaviors, whereas others have proposed that stress partially mediates the effects of poor sleep on dietary choices and emotional eating [51,59,60]. However, the simultaneous retention of both variables in the final multivariable model indicates that each contributes unique explanatory information regarding EE. Although the underlying mechanisms could not be examined directly, the observed findings are consistent with theoretical models linking perceived stress and sleep quality to EE through complex behavioral and physiological pathways. Stress primarily affects eating behavior through activation of the HPA axis, cortisol secretion, and emotion-focused coping mechanisms [63], whereas poor sleep quality may influence eating behavior through alterations in leptin and ghrelin secretion, impaired executive functioning, reduced inhibitory control, increased emotional reactivity, and enhanced responsiveness to food-related reward cues [64]. The coexistence of these mechanisms may explain why students simultaneously experiencing elevated stress and poor sleep quality appear particularly vulnerable to EE and other maladaptive eating behaviors.

An additional finding of the present study was the association between nationality and EE. Although nationality was included in the multivariable model, the category of non-Greek students represented a heterogeneous population originating from different cultural backgrounds. Cultural differences in dietary practices, food availability, body image perceptions, smoking habits, physical activity patterns, academic expectations, and coping responses to stress may influence EE behaviors. Furthermore, international students may experience unique stressors related to acculturation, language barriers, social integration, and adaptation to a new educational environment. Therefore, the observed association between nationality and EE should be interpreted cautiously, as residual confounding from unmeasured cultural and socioeconomic factors cannot be excluded. We found that Greek students had higher odds of EE than non-Greek students, which seems somewhat counterintuitive compared with what might be expected for international students. However, the relatively small and heterogeneous non-Greek subgroup may partly explain this finding and should be interpreted cautiously. Future studies should collect more detailed information regarding country of origin, ethnicity, and acculturation-related characteristics to better understand the role of cultural background in emotional eating behaviors.

### Strengths and Limitations

The present study possesses several important strengths that enhance the robustness and relevance of its findings. First, to the best of our knowledge, it represents one of the largest investigations conducted among Greek university students examining the relationship between EE, perceived stress, and sleep quality. The relatively large sample size provided adequate statistical power to detect meaningful associations and allowed the simultaneous evaluation of numerous sociodemographic, academic, lifestyle, anthropometric, and behavioral variables. Second, the study adopted a comprehensive analytical approach by incorporating perceived stress, sleep quality, physical activity, smoking status, academic characteristics, and multiple indicators of adiposity within the same multivariable model. This enabled the identification of independent associations while minimizing the potential influence of confounding factors. Third, the use of validated and widely employed assessment instruments increased the reliability and comparability of the findings with those of previous studies. Fourth, the inclusion of both general and central obesity indicators (BMI, WC, and WHR) provided a more detailed evaluation of the relationship between EE and body composition than studies relying solely on BMI. Finally, the adequacy of the ordinal logistic regression model was confirmed through diagnostic testing, including verification of the proportional odds assumption and overall model fit, supporting the validity of the reported associations.

Despite these strengths, several limitations should be considered when interpreting the findings. The cross-sectional design precludes the establishment of causal relationships and prevents determination of the temporal sequence between EE, perceived stress, sleep quality, and the other associated factors. Consequently, bidirectional relationships remain possible; for example, EE may both result from and contribute to increased psychological distress and sleep disturbances. Moreover, because of the cross-sectional nature of the study, temporal relationships and causal inferences cannot be established. Consequently, the observed associations should not be interpreted as evidence that perceived stress or sleep quality directly influence EE. Furthermore, the biological and behavioral mechanisms potentially underlying these associations were not directly assessed and therefore remain speculative. In addition, the study relied on self-reported questionnaire data, which may be subject to recall bias, reporting bias, and social desirability effects. Although a broad range of potential confounders was included in the analysis, residual confounding from unmeasured variables, such as dietary intake patterns, chronotype, social support, personality traits, coping styles, or other mental health conditions, cannot be excluded. Furthermore, because the study population consisted exclusively of university students, caution is warranted when generalizing the findings to non-student young adults or other age groups.

Moreover, although nationality was included as a covariate in the multivariable regression model to account for its potential confounding effect, residual confounding related to unmeasured cultural and socioeconomic characteristics cannot be excluded. We have therefore acknowledged this issue as a limitation of the present study and suggested that future investigations collect more detailed information on countries of origin, ethnicity, acculturation, and cultural background to better elucidate these relationships.

We also acknowledge that the use of a non-probability sampling strategy may have introduced selection bias, as participation was voluntary and students who were more interested in health-related topics or more willing to participate in research may have been overrepresented. Thus, although recruitment was conducted across ten geographically diverse regions of Greece, the representativeness of the sample relative to the entire Greek university student population cannot be guaranteed. Consequently, caution is warranted when extrapolating the present findings to all university students in Greece. To overcome this limitation, future nationwide studies employing probability-based sampling methods would strengthen the representativeness and external validity of the findings.

In addition, the comparison of our study sample with the broader university student population would provide useful information regarding representativeness. However, comprehensive and contemporaneous data describing the sociodemographic characteristics of the overall Greek university student population, as well as those of university students within each participating region, were not available. Consequently, potential differences between our study sample and the target population cannot be excluded, and caution should be exercised when generalizing the findings.

Furthermore, although perceived stress and sleep quality emerged as strong independent correlates of EE, the underlying biological and behavioral mechanisms could not be directly assessed within the present study. Therefore, prospective longitudinal studies and intervention trials are needed to clarify causal pathways and determine whether improvements in stress management and sleep quality can lead to sustained reductions in EE behaviors among university students.

Finally, depression and anxiety have also been identified as important psychological correlates of EE. In a previous study conducted by our research group among a moderately overlapping Greek university students, both depressive and anxiety symptoms were significantly associated with emotional eating behavior [65]. Although these variables were beyond the scope of the present investigation, their potential interplay with perceived stress and sleep quality should be acknowledged. Future studies employing comprehensive psychological assessments and longitudinal designs may help clarify the relative contribution and interrelationships of these factors.

## 5. Conclusions

The present findings strengthen and extend the existing literature supporting evidence that stress and sleep quality may be associated with EE behavior in university students. The magnitude and persistence of these associations after extensive adjustment for potential confounders underscore the importance of considering psychological distress and sleep health as central components of EE prevention and intervention strategies. These findings suggest that interventions focusing exclusively on dietary education may be insufficient unless they are accompanied by programs targeting stress reduction, emotional regulation, sleep hygiene, and broader psychological well-being.

Future research should employ longitudinal designs to better elucidate the temporal directionality and potential bidirectional relationships underlying these associations. Furthermore, the inclusion of objective behavioral, physiological, and biological measures, alongside psychosocial assessments, would enhance mechanistic understanding of emotional eating. Despite inherent limitations, the present study contributes to the expanding literature by reinforcing the conceptualization of EE as a critical interface between mental health and lifestyle-related behaviors in young adult populations.

## Figures and Tables

**Table 1:** Descriptive statistics of the enrolled university students.

Variables (*n* = 1297)	Descriptive Statistics
**Age (mean ± SD; years)**	20.5 ± 2.5
**Gender (*n*, %)**	
Male	564 (43.5%)
Female	733 (56.5%)
**Nationality (*n*, %)**	
Greek	1063 (82.0%)
Other	234 (18.0%)
**Type of residence (*n*, %)**	
Urban	680 (52.4%)
Rural	617 (47.6%)
**Family economic status (*n*, %)**	
Low	523 (40.3%)
Medium	484 (37.3%)
High	290 (22.4%)
**Living status (*n*, %)**	
Living with family	723 (55.7%)
Living alone	574 (44.3%)
**Parents marital status (*n*, %)**	
No divorced	881 (67.9%)
Divorced	416 (32.1%)
**Smoking status (*n*, %)**	
No smokers	875 (67.5%)
Smokers	422 (32.5%)
**Type of Studies (*n*, %)**	
Biomedical studies	710 (54.7%)
Other studies	587 (45.3%)
**Years of Studies (*n*, %)**	
One year	333 (25.7%)
Two years	316 (24.4%)
Three years	318 (24.5%)
Four years	330 (25.4%)
**Academic performance (*n*, %)**	
Good	613 (47.2%)
Very good	412 (31.8%)
Excellent	272 (21.0%)
**Employment status (*n*, %)**	
Employee	462 (35.6%)
No employee	835 (64.4%)
**Physical activity (*n*, %)**	
Low	648 (50.0%)
Medium	358 (27.6%)
High	291 (22.4%)
**BMI (*n*, %)**	
Normal weight	740 (52.1%)
Overweight	426 (32.8%)
Obese	131 (10.1%)
**WC (*n*, %)**	
Normal	799 (61.6%)
Increased risk	339 (26.1%)
High risk	159 (12.3%)
**WHR (*n*, %)**	
Low risk	852 (65.7%)
Moderate risk	338 (26.1%)
High risk	107 (8.2%)
**Perceived Stress (*n*, %)**	
Low	671 (51.7%)
Moderate	319 (24.6%)
High	307 (23.7%)
**Sleep Quality (*n*, %)**	
Adequate	848 (65.4%)
Inadequate	449 (34.6%)
**TEFQ-R18 (mean ± SD)**	34.8 ± 21.5
**TEFQ-R18 (Tertiles)**	
Low EE (Q1, *n*%)	433 (33.4%)
Moderate EE (Q2, *n*%)	432 (33.3%)
High EE (Q3, *n*%)	432 (33.3%)

**Table 2:** Associations of Emotional Eating (EE) with sociodemographic, anthropometric, lifestyle and psychological factors of the enrolled university students.

Variables (*n* = 1279)	Emotional Eating (EE) *			
	Low 433 (33.4%)	Medium 432 (33.3%)	High 432(33.3%)	*p*-Value
**Age (mean ± SD; years)**	20.5 ± 2.4	20.4 ± 2.5	20.3 ± 2.5	*p* = 0.4326
**Gender (*n*, %)**				*p* ˂ 0.0001
Male	233 (41.3%)	161 (28.6%)	170 (30.1%)	
Female	200 (27.3%)	271 (37.0%)	262 (35.7%)	
**Nationality (*n*, %)**				*p* = 0.0001
Greek	328 (30.9%)	378 (35.6%)	357 (33.6%)	
Other	105 (44.9%)	54 (23.1%)	75 (32.0%)	
**Type of residence (*n*, %)**				*p* ˂ 0.0001
Urban	268 (39.3%)	222 (32.7%)	190 (29.9%)	
Rural	165 (26.8%)	210 (34.0%)	242 (39.2%)	
**Family economic status (*n*, %)**				*p* = 0.131
Low	176 (33.7%)	165 (31.6%)	182 (33.7%)	
Medium	169 (34.9%)	152 (31.4%)	163 (37.7%)	
High	88 (30.3%)	115 (31.4%)	87 (33.7%)	
**Living status (*n*, %)**				*p* = 0.1219
Living with family	207 (28.6%)	272 (37.6%)	244 (33.8%)	
Living alone	226 (39.4%)	160 (27.9%)	188 (32.8%)	
**Parents marital status (*n*, %)**				*p* = 0.1054
No divorced	279 (31.7%)	324 (36.8%)	278 (31.6%)	
Divorced	154 (37.0%)	108 (26.0%)	154 (37.0%)	
**Smoking status (*n*, %)**				*p* ˂ 0.0001
No smokers	332 (37.9%)	302 (34.5%)	241 (27.6%)	
Regular smokers	101 (23.9%)	130 (30.8%)	191 (45.3%)	
**Type of Studies (*n*, %)**				*p* ˂ 0.0001
Biomedical studies	188 (26.5%)	285 (40.1%)	237 (33.4%)	
Other studies	245 (41.6%)	147 (25.1%)	195 (33.3%)	
**Years of Studies (*n*, %)**				*p* ˂ 0.0001
One year	99 (29.7%)	53 (15.9%)	181(54.4%)	
Two years	108 (34.2%)	99 (31.3%)	109 (34.5%)	
Three years	95 (29.9%)	140 (44.0%)	83 (26.1%)	
Four years	131 (39.7%)	140 (44.2%)	59 (17.9%)	
**Academic performance (*n*, %)**				*p* ˂ 0.0001
Good	209 (34.1%)	158 (25.8%)	246 (40.1%)	
Very good	124 (30.1%)	176 (42.7%)	112 (27.2%)	
Excellent	100 (36.8%)	98 (36.0%)	74 (27.2%)	
**Employment status (*n*, %)**				*p* = 0.9031
No employee	282 (33.8%)	278 (33.3%)	275 (32.9%)	
Employee	151 (32.7%)	154 (33.3%)	157 (34.0%)	
**Physical activity (*n*, %)**				*p* ˂ 0.0001
Low	154 (23.8%)	228 (35.2%)	266 (41.0%)	
Medium	160 (44.7%)	104 (29.0%)	94 (26.3%)	
High	119 (40.9%)	100 (34.4%)	72 (24.7%)	
**BMI (*n*, %)**				*p* ˂ 0.0001
Normal weight	316 (42.7%)	331 (44.7%)	93 (12.6%)	
Overweight	98 (23.0%)	81 (19.0%)	247 (58.0%)	
Obese	19 (14.5%)	20 (15.3%)	92 (70.2%)	
**WC (*n*, %)**				*p* ˂ 0.0001
Normal	330 (41.3%)	339 (42.4%)	130 (16.3%)	
Increased risk	74 (21.8%)	60 (17.7%)	205 (60.5%)	
High risk	29 (18.2%)	33 (20.8%)	97 (61.0%)	
**WHR (*n*, %)**				*p* ˂ 0.0001
Low risk	301 (35.3%)	302 (35.5%)	249 (29.2%)	
Moderate risk	93 (27.5%)	116 (34.3%)	129 (38.2%)	
High risk	39 (36.4%)	14 (13.1%)	54 (50.5%)	
**Perceived Stress (*n*, %)**				*p* ˂ 0.0001
Low	341 (50.8%)	192 (28.6%)	138 (20.6%)	
Moderate	63 (19.7%)	175 (54.9%)	81 (25.4%)	
High	29 (9.4%)	65 (21.2%)	213 (69.4%)	
**Sleep Quality (*n*, %)**				*p* ˂ 0.0001
Adequate	345 (40.7%)	263 (31.0%)	240 (28.3%)	
No adequate	88 (19.6%)	169 (37.6%)	192 (47.8%)	

* Percentages are calculated by row and represent the proportion of participants within each category of the explanatory variable.

**Table 3:** Multivariate ordinal logistic regression model of factors associated with emotional eating (EE).

Variable	Category	Adjusted OR *	95% CI **	*p*-Value
**Gender**	Female vs. Male	1.43	1.11–1.83	0.0043
**Nationality**	Greek vs. Other	1.18	1.06–1.30	0.0022
**Type of residence**	Rural vs. Urban	1.53	1.19–1.97	0.0009
**Smoking status**	Regular smokers vs. No smokers	1.68	1.27–2.23	0.0003
**Type of Studies**	Biomedical studies vs. Other studies	1.23	1.07–1.42	0.0046
**Years of Studies**	1st + 2nd vs. 3rd + 4th	1.22	1.07–1.39	0.0032
**Academic performance**	Good vs. Very good	1.15	0.88–1.51	0.3005
	Good vs. Excellent	1.24	0.91–1.69	0.1659
**Physical activity**	Low vs. Medium	1.38	1.08–1.75	0.0078
	Low vs. High	2.12	1.41–3.22	0.0004
**BMI**	Overweight vs. Normal	1.95	1.33–2.87	0.0007
	Obese vs. Normal	2.10	1.45–3.05	0.0001
**WC**	Increased risk vs. Normal	1.31	0.83–2.08	0.2452
	High risk vs. Normal	1.93	1.33–2.80	0.0005
**WHR**	Moderate risk vs. Normal	1.24	0.82–1.89	0.3107
	High risk vs. Normal	2.05	1.42–2.93	0.0001
**Perceived Stress**	Moderate vs. Low	2.01	1.41–2.87	0.0001
	High vs. Low	2.86	1.68–4.85	0.0001
**Sleep Quality**	Inadequate vs. Adequate	3.08	1.75–5.42	0.0001

* Odds Ratio: OR; ** CI: Confidence Interval.

## Data Availability

Data is available upon request to the corresponding author.

## Supplementary Materials

The following supporting information can be downloaded at: https://www.mdpi.com/article/10.3390/nu18152434/s1, Table S1: Spearman correlation coefficients among emotional eating (EE), perceived stress (PSS), and sleep quality (PSQI) scores as continuous variables; Table S2. Multiple linear regression.
