# Supplementary material for: Associations of Emotional Eating with Perceived Stress and Sleep Quality Among University Students: A Nationwide Cross-Sectional Study
Source: Nutrients. 2026 Jul 25;18(15):2434. doi: 10.3390/nu18152434 (PMC13468451; doi:10.3390/nu18152434)
Supplement: Supplementary file 1 [file nutrients-18-02434-s001.zip › nutrients-4416544-supplementary.pdf]

Supplementary Table S1. Spearman correlation coefficients among emotional eating (EE), perceived stress (PSS), and sleep quality (PSQI) scores as continuous variables

Variable	EE Score	PSS score	PSQI score
EE Score	1.00	0.46*	0.41*
PSS score	0.46*	1.00	0.43*
PSQI score	0.41*	0.43*	1.00

*$p < 0.0001$

Supplementary Table S2. Multiple linear regression

Variable	β Coefficient (SE)	Standardized β	p-value
PSS score	0.86 (0.06)	0.40	$p < 0.001$
PSQI score	1.15 (0.11)	0.29	$p < 0.001$
